# Five Surfaces Treated with d-Tetramethrin plus Acetamiprid for the Management of *Tenebrio molitor* and *Alphitobius diaperinus*: Which Is the Best?

**DOI:** 10.3390/insects14050452

**Published:** 2023-05-11

**Authors:** Nickolas G. Kavallieratos, Erifili P. Nika, Penelope D. Gounari

**Affiliations:** Laboratory of Agricultural Zoology and Entomology, Department of Crop Science, Agricultural University of Athens, 75 Iera Odos Str., 11855 Athens, Greece; erifilinika@aua.gr (E.P.N.);

**Keywords:** plastic, glass, metal, wood, ceramic, surface treatment, yellow mealworm, lesser mealworm

## Abstract

**Simple Summary:**

This study evaluated the minimum as well as the maximum label dose of d-tetramethrin plus acetamiprid applied on plastic, glass, metal, wood, and ceramic surfaces, against adults of both species, in terms of immediate and delayed mortality. All *T. molitor* died after a 7-day exposure to the maximum dose at all tested surfaces when food was absent. In the presence of food, only glass-, metal-, and ceramic-treated surfaces killed all adults at the same dose. Even the minimum dose provided 100% mortality on glass, metal, and ceramic surfaces in both food scenarios. In the case of *A. diaperinus*, only the maximum dose caused the death of all exposed individuals on glass (with food) and on glass and plastic (no food). Overall, the maximum dose of d-tetramethrin plus acetamiprid is effective against the tested species on certain types of surfaces.

**Abstract:**

*Tenebrio molitor* L. (Coleoptera: Tenebrionidae) and *Alphitobius diaperinus* Panzer (Coleoptera: Tenebrionidae) are two common tenebrionids occurring in grain storages. In this study, we assessed the immediate and delayed mortalities caused by d-tetramethrin plus acetamiprid on five different surfaces, i.e., plastic, glass, metal, wood, and ceramic, against adults of the two species. The tests included two label doses of the insecticide (minimum and maximum) and two food scenarios (food and no food). Generally, the maximum dose was more efficient than the minimum dose, and the presence of food resulted in lower observed mortalities than when food was absent. *Tenebrio molitor* was more susceptible than *A. diaperinus*, at all dose, food, and surface scenarios. At delayed bioassays, both doses killed all *T. molitor* on plastic, while on wood, mortality ranged between 80.6 and 100.0%, regardless of the food scenario. Concerning *A. diaperinus*, delayed mortalities ranged among treated surfaces, food scenarios, and dose from 58.3 to 100.0%. The insecticide killed the most individuals when it was treated on glass, while when it was applied on wood caused the death of the least individuals. Concerning plastic, metal, and ceramic surfaces, no general trend was observed. The maximum dose of the tested insecticide provides elevated mortalities for both species when food is absent.

## 1. Introduction

*Tenebrio molitor* L. (Coleoptera: Tenebrionidae) is an important and noxious stored-product insect related to poor storage conditions and hygiene [1]. Apart from the direct consumption of the grains during their storage, *T. molitor* contaminates the products with its excrements and dead body particles, decreasing food quality [2]. This species can provoke allergic reactions, such as respiration problems and eczema, commonly appearing in people handling storage foods [3,4,5,6,7,8]. Lately, *T. molitor* has been in the spotlight since it is used by the food and recycling industry [9,10,11,12,13,14,15,16,17,18,19]. Its insect powder enhances food properties such as nutritional value, crispness, taste, and digestibility [9,12,16,18]. Apart from human nutrition, this species is used as feed for a plethora of organisms such as fish, reptiles, birds, and crustaceans [20,21,22,23,24]. In addition, recent studies revealed that *T. molitor* extracts display antiproliferative, antimicrobial, antifreeze, antithrombotic, preservative, and healing properties [25,26,27,28,29].

One other harmful insect worldwide is *Alphitobius diaperinus* Panzer (Coleoptera: Tenebrionidae) [20,30]. It infests more than 70 different stored products [30], while at the same time is really prevalent in farms where livestock is raised feeding on dead animals such as mice and birds [1]. This species causes a huge problem at poultry facilities [20,30,31,32]. *Alphitobius diaperinus* has a short developmental period at optimal conditions and is long-lived [1]. Due to the continuous food presence and the prevailing temperature range at poultry houses, *A. diaperinus* completes its life circle in short periods of time, supporting large populations [33]. Furthermore, chickens consume *A. diaperinus* individuals that may be infected by viruses, bacteria, protozoa, and fungi, since this species is a vector of multiple pathogens [34,35,36,37,38,39]. These microorganisms can also harm humans, causing symptoms such as vomiting, fever, diarrhea, and cramps in the abdomen [40,41,42]. Employees exposed to these insects can display allergic reactions such as asthma, rhinitis, urticaria, conjunctivitis, and angioedema [43,44,45]. Apart from the health problems it provokes, it damages the structure of facilities by consuming the insulation of walls and ceilings [46].

Given the fact that both pests are enemies of major importance, several insecticides, mainly synthetic chemicals, i.e., neonicotinoids, organophosphates, pyrethroid compounds, and synergized pyrethrins, as well as insecticides of natural origin, i.e., essential oils, diatomaceous earths, and nanoemulsions, have been utilized over the years in order to handle possible invasions [2,47,48,49,50,51,52,53,54,55,56,57,58]. Among all these studies, only a few of them examined the effectiveness of the insecticides applied on surfaces. In particular, Athanassiou et al. [48] studied the efficacy of α-cypermethrin and thiamethoxam applied on concrete against *T. molitor* adults, while Kavallieratos et al. [56,57] examined chlorfenapyr and deltamethrin sprayed on concrete surfaces against *A. diaperinus* adults. In the case of *A. diaperinus*, the tests investigated the impact of the presence/absence of food on the treated surfaces [56,57], whereas, regarding *T. molitor*, all tests included food [48]. Furthermore, Lyons et al. [49] experimented with permethrin and β-cyfluthrin treated on pressure-treated wood, particle board (wood-chip-type), and concrete to control *A. diaperinus* adults.

The formulation consisting of the two active ingredients (a.i.s) (i) d-tetramethrin (pyrethroid insecticide) and (ii) acetamiprid (neonicotinoid insecticide), and the insecticide synergist piperonyl butoxide, is used for surface treatments [55,59,60,61]. Previously, the efficacy of this formulation was assessed against small/large larvae of *A. diaperinus*, with/without food, on concrete surfaces [55]. This mixture of a.i.s was more effective against small larvae than large larvae, but its effectiveness against adults of the species is yet to be discovered.

Given that insect farms, storage units, and poultry houses consist of numerous surfaces [20,62], there are no data available considering the impact of plastic, glass, metal, wood, and ceramic surfaces on the effectiveness of d-tetramethrin plus acetamiprid against adult individuals of *T. molitor* and *A. diaperinus*, to reveal the optimal surface for treatment. Therefore, in this study, the immediate and delayed mortality of the minimum and maximum label dose of the aforementioned formulation was assessed. To delve deeper, the parameter of food presence/absence was taken into consideration.

## 2. Materials and Methods

### 2.1. Insects and Food

The two tested tenebrionids were bred at the Laboratory of Agricultural Zoology and Entomology (Agricultural University of Athens, Athens, Greece). Both were laboratory cultures derived from Laboratory of Agricultural Entomology (Benaki Phytopathological Institute, Kifissia, Attica, Greece). The rearing mediums were wheat bran with potato, or apple cuts for extra moisture, for *T. molitor* and *A. diaperinus*, respectively. Both species were kept under the following conditions: 30 °C, 65% relative humidity (RH), and complete light absence [53,63,64]. The participating tenebrionids were taken randomly from the colonies. The adults were younger than 14 or 7 days old (days since their emergence from pupae) for *T. molitor* and *A. diaperinus*, respectively.

### 2.2. Insecticide

Dobol^®^ EC was used to treat the tested surfaces. This insecticide consists of d-tetramethrin [3,4,5,6-tetrahydrophthalimidomethyl (1RS)-cis-trans-chrysanthemate] (2.5% *w*/*v*) plus acetamiprid [N-[(6-chloropyridin-3-yl)methyl]-N′-cyano-N-methylethanimidamide] (5% *w*/*v*), and piperonyl butoxide (3,4-methylenedioxy-6-propylbenxyl n-butyl diethyleneglycol ether) (10% *w*/*v*) a.i.s (Société Kwizda France, Marly le Roi, France). There are two label doses tested for surface applications: (i) minimum (min), i.e., 0.0001 mL formulation/cm^2^, and (ii) maximum (max), i.e., 0.0002 mL formulation/cm^2^.

### 2.3. Bioassays

For the experiment, surfaces of plastic, glass, metal, wood, and ceramic were treated with the aforementioned doses of insecticide. For this purpose, three replicates of Petri dishes, consisting of three subreplicates each, were prepared. The dishes were plastic (in the cases of plastic, metal, wood, and ceramic surfaces) or glass (in the case of glass surfaces) and had the following dimensions: 1.5 cm height; 8 cm diameter. Metal, wood, and ceramic surfaces were cut from galvanized metal sheets (1 mm thick), pieces of plywood (4 mm thick), and ceramic tiles (6 mm thick), respectively, purchased from local stores. The type of plastic that dishes were made from was polystyrene. Concerning plastic and glass, insecticides were applied directly to the dishes, while metal, wood, and ceramic pieces were cut to fit perfectly (<50.27 cm^2^ area) into plastic dishes. The internal vertical dish surface of each dish (plastic or glass) was coated with polytetrafluoroethylene, acquired from Sigma-Aldrich Chemie GmbH (Taufkirchen, Germany). This coat prevented tenebrionids from escaping. Lids had a circular cut (1.5 cm diameter); thus, dishes were fully aerated. This cut was covered with cloth. Each surface/dish was sprayed with 1 mL volume that contained the desired quantity of each tested dose. The spraying was conducted with an airbrush (AG-4 Mecafer S.A., Valence, France) in a fine mist. Afterward, a balance (Precisa XB3200D, Alpha Analytical Instruments, Gerakas, Greece) was used to weigh quantities of 0.5 g, derived from the diet of the tested tenebrionids, which was finally spread into the treated dishes. The above procedure was repeated, but this time no diet was introduced into the dishes. As controls, additional series of dishes (of each type of surface) were sprayed with distilled water. The control series were repeated twice, once with and once without diet. Two hours after spraying, 10 individuals were inserted in each dish and transferred into 30 °C/65% RH incubators, for the entire duration of the experiments. After 1, 3, 5, and 7 days, mortality of both tenebrionid species was counted with a stereomicroscope (Olympus SZX9, Bacacos S.A., Athens, Greece). For the insect inspection, a brush was used to gently poke the individuals. No movement detection meant that insects were dead. After a 7-day exposure to the treated surfaces, all living insects were transferred to non-treated surfaces for 7 additional days to evaluate delayed mortality. Adults that were exposed to a certain type of treated surface were put into dishes with the same type of non-treated surface. Furthermore, adults exposed to the insecticide with diet into the dishes were conveyed to non-treated dishes with new 0.5 g diet. Similarly, adults exposed to the insecticide with no diet on the dishes were conveyed to non-treated dishes with no diet. This procedure was followed to evaluate delayed mortality for controls. Mortalities were counted as above, at the end of the experiment.

### 2.4. Data Analysis

Both mortality (immediate and delayed) control values were lower than 5% for the two tenebrionid species. Therefore, no corrections were made to the mortality data, but they were log (x + 1) converted in advance of the analysis to ensure the variance normalization [65,66]. Each species was submitted to a separate repeated-measures multivariate analysis of variance (MANOVA) [67]. The repeated factor was exposure, response variable was mortality, and main effects were dose, surface, and presence/absence of food. Associated interactions (of main effects) were incorporated into the analysis. For each species, delayed mortality data were separately analyzed with a two-way ANOVA, separately for each tested dose. The response variable was mortality, whereas surface and food presence/absence were the main effects. The Tukey–Kramer test (HSD) or the two-tailed *t* test assorted means at 0.05 level of significance were used [68]. The JMP 16.2 software was used for all analyses [69].

## 3. Results

### 3.1. Immediate Mortality of Tenebrio molitor

Between exposure intervals, all main effects and food × surface interaction were significant (Table 1). Concerning the intervals within exposure, all main effects were significant. Exposure × food × surface and exposure × dose × food × surface interactions were also significant. On the first day of the experiment, mortalities of the minimum dose of d-tetramethrin plus acetamiprid ranged from 0.0% (applied on metal) to 8.9% (applied on glass) when food was present (Table 2). By the end of the week, this dose killed all *T. molitor* adults when applied on glass, metal, and ceramic surfaces. Meanwhile, plastic and wood surfaces treated with this dose provided 96.7 and 87.8% mortality, respectively, in the same food scenario. The minimum dose caused significantly variable mortalities on the first day of exposure (1.1–37.8%) on surfaces without food. After 5 days, the treated glass and ceramic surfaces caused the death of all exposed adults. By the end of the experiments, plastic and metal killed all *T. molitor* individuals, while wood provided 92.2% mortality at the same exposure interval. Glass and ceramic surfaces treated with the maximum dose of the insecticide caused the death to all exposed adults after a 5-day exposure in the presence of food. The same dose and food scenario killed all adults exposed to metal after 7 days, while plastic and wood-treated surfaces reached 97.8 and 94.4% mortality, respectively. Similarly, the maximum dose, when food was absent, killed all individuals exposed to treated glass and ceramic surfaces after 5 days. All *T. molitor* adults were dead on treated plastic, metal, and wood 7 days post-exposure in the same food and dose scenario.

### 3.2. Immediate Mortality of Alphitobius diaperinus

Between and within exposure, all main effects were significant (Table 1). Dose × surface (between), exposure × dose × surface, and exposure × dose × food × surface interactions (within) were also significant. When *A. diaperinus* adults were exposed to the minimum dose of d-tetramethrin plus acetamiprid in the presence of food, no dead individuals were detected on the 1st day of the experiments on metal and wood, while treated ceramic, plastic, and glass surfaces provided mortality ranging from 1.1 to 17.8% (Table 3). After 7 days of exposure, this dose killed 53.3% (applied on wood) to 97.8% (applied on glass) in the same food scenario. When the minimum dose of this insecticide was applied in the absence of food, mortalities ranged between 0.0% (applied on metal) and 18.9% (applied on glass) after 1 day of exposure and between 63.3% (applied on wood) and 98.9% (applied on glass) 7 days post-exposure. The maximum dose of d-tetramethrin plus acetamiprid with food provided complete mortality (100.0%) when it was applied on glass, 97.8% on plastic, 87.8% on ceramic, 84.4% on metal, and 80.0% on wood surfaces 7 days post-exposure. The same dose in the absence of food provoked the death of all individuals that were exposed to treated plastic and glass surfaces by the end of the experiments. When the insecticide was applied on the other three types of surfaces, it killed 87.8% (wood), 92.2% (ceramic), and 97.8% (metal) after a 7-day exposure.

### 3.3. Delayed Mortality of Tenebrio molitor

All remaining *T. molitor* individuals died after their exposure to the minimum dose of d-tetramethrin plus acetamiprid treated on plastic in the presence of food (Table 4). The same dose treated on wood killed 80.6% and 100.0% of the remaining adults in the presence or absence of food, respectively. Concerning the maximum dose of the insecticide, all remaining individuals died on both plastic and wood surfaces when food was present.

### 3.4. Delayed Mortality of Alphitobius diaperinus

All the remaining *A. diaperinus* adults died after their exposure to plastic and glass surfaces treated with the minimum dose of d-tetramethrin plus acetamiprid for both food and no-food scenarios (Table 5). Concerning metal, wood, and ceramic surfaces, the mortality of remaining adults reached 91.7, 84.3, and 84.4% in the presence of food, and 81.9, 91.3, and 89.3% in the absence of food, respectively. Regarding the maximum dose of the insecticide, all remaining *A. diaperinus* died on plastic and metal surfaces in the presence of food, as well as on wood surfaces in the absence of food. The scenarios metal/no food, wood/food, and ceramic/food caused the death of 75.0, 92.9, and 92.9% of the remaining adults. The lowest delayed mortality was noted in the case of ceramic surfaces, when food was absent, not exceeding 58.3% at the end of the assays.

## 4. Discussion

The findings of the current study revealed that there are significant differences in the impact of d-tetramethrin plus acetamiprid depending on the type of surfaces they were applied to. In general, the insecticide applied on glass resulted in the most elevated mortality rates, while when it was applied on wood, it killed the fewest adult individuals, regardless of food/no food scenario and insect species, on both immediate and delayed bioassays. There was no general trend among the other three types of surfaces, i.e., metal, plastic, and ceramic. However, treatments on metal and ceramic caused higher mortality levels than plastic surfaces. This was evident for both immediate and delayed mortalities for *T. molitor* adults, given that there was no delayed mortality in the case of metal and ceramic. The variable structural properties of the tested surfaces, i.e., the existence of pores, may cause the observed differential insecticidal efficacy. Glass, metal, and ceramic are non-porous surfaces, while plastic, followed by wood, are more porous surfaces [70,71,72]. Arthur [73] understood the significance of porous and non-porous surfaces, and how they affected the efficacy of the insecticides applied to them. The author noticed that when concrete was sealed, the applied insecticides had better residual efficacy than non-sealed concrete, i.e., concrete with pores [73]. Similarly to our results, Arthur [74] reported that deltamethrin treated on ceramic tile had higher efficacy than when it was treated on wood surfaces. In a previous study, Vojoudi et al. [72] applied abamectin, chlorpyrifos, and deltamethrin on paper, plastic, ceramic, and glass surfaces. The authors found that higher doses of insecticide were needed to kill 90% of *Tribolium castaneum* (Herbst) (Coleoptera: Tenebrionidae) on paper < plastic < ceramic < glass. In a more recent study, Arthur et al. [75] tested deltamethrin and cyfluthrin against young/old larvae of *Trogoderma granarium* Everts (Coleoptera: Dermestidae). The authors applied the insecticides on metal, vinyl flooring tile, wood, painted wood, and concrete surfaces. Treated metal provided the highest mortalities for a period of three months [75]. Combining the findings of this study with the previous studies, the type of surface should be taken into consideration when insecticidal treatments will be followed for the management of *T. molitor* and *A. diaperinus* adults. Furthermore, whether more porous surfaces, other than concrete, should be sealed before treatment [73] merits investigation.

In all tested scenarios, *T. molitor* adults were more susceptible than *A. diaperinus* adults. This was documented before in several studies, since there is no general trend in tenebrionids concerning their developmental stage susceptibility to insecticides [54,76]. For example, *T. molitor’s* susceptible developmental stage is the adult stage, while larvae are more tolerant [53]. However, this does not apply to *A. diaperinus*, since larvae are its susceptible developmental stage, and adults are more tolerant than larvae [56,57]. Susceptibility differences among species and their developmental stages occur because of the variability of their cuticle’s lipid composition and/or variety, their setae, their morphology, and their behavior [77,78,79,80,81,82,83,84,85,86,87,88,89,90,91,92,93,94]. In a more recent study, Kavallieratos et al. [55] treated concrete surfaces with d-tetramethrin plus acetamiprid against *A. diaperinus* small and large larvae. The formulation killed more small than large larvae, while the presence of food caused a significant reduction in the efficacy of the insecticide [55]. Total mortality was observed only in the case of small larvae [55]. These are rather important findings since d-tetramethrin plus acetamiprid applied on porous surfaces is not able to suppress the susceptible developmental stage of *A. diaperinus*, i.e., larvae [55], while the same insecticide treated on non-porous surfaces (glass) killed all the tolerant individuals, i.e., adults. These results prove the significance of the comprehensive organization of pest management strategies to choose the best surface for insecticidal application, depending on the target pest and/or developmental stage.

One other important factor concerning the efficacy of d-tetramethrin plus acetamiprid was the presence/absence of food on treated surfaces, in terms of immediate and delayed mortality. Generally, food presence resulted in lower observed mortality rates at both doses, regardless of the insect species. Similarly, Kavallieratos et al. [55,56,57] noticed that food on treated concrete reduced mortality levels caused by chlorfenapyr, deltamethrin, etofenprox, and d-tetramethrin plus acetamiprid, against small/large larvae and adults of *A. diaperinus*. Food is considered an obstacle since it can prevent the contact of the insect with the treated surface, but also because it can absorb or even degrade the insecticide [95,96,97,98,99,100,101,102,103]. As a result, intensive cleaning is suggested to eliminate the possibility of the insecticidal efficacy reduction caused by food prior to the application, as well as during the storage period [99,104,105].

Concerning d-tetramethrin plus acetamiprid, this is a mixture of a.i.s that has been scarcely examined. After an extensive search into the international bibliography, only Kavallieratos et al. [55] and Kavallieratos and Boukouvala [106] have examined this mixture on *A. diaperinus* and *T. granarium*, respectively. The authors revealed the elevated efficacy on both adults and larvae of *T. granarium*, reaching 100.0% and 84.4% mortality, respectively [106]. This was an important finding since *T. granarium* larvae are very tolerant. Taking into account our findings and those of the aforementioned studies, we can conclude that this mixture of a.i.s has the potential concerning stored-product pest management in storages. It can also be assumed that piperonyl butoxide, as an insecticide synergist that enhances the insecticidal properties of d-tetramethrin and acetamiprid [59,107], plays a significant role in the elevated efficacy of this mixture in this study, compared with the insecticidal properties of these a.i.s in former studies [108,109,110,111,112,113,114,115]. Previous research has revealed that the incorporation of piperonyl butoxide into pyrethroid insecticides did not alter the susceptibility of the susceptible strain of *A. diaperinus* [113]. It should be noted that former studies revealed the antifeedant and repellent effects of tetramethrin [109,110]. This can partially explain the lower delayed mortalities observed in no-food scenarios.

## 5. Conclusions

To conclude, *A. diaperinus* adults were more tolerant than *T. molitor* adults, regardless of insecticidal dose, surface, and food/no food scenarios. When both doses were applied on wood resulted in the lowest mortality levels against both species, while treated glass provided the most elevated mortality, in all tested food/no food scenarios. No trend was revealed during this study concerning the suitability of treated plastic, metal, and ceramic surfaces against these tenebrionids in the absence or presence of food. Food absence resulted in elevated efficacy, contrary to the presence of food. Overall, d-tetramethrin plus acetamiprid is effective against both tested species when they are treated on several types of surfaces. Whether there are ways to enhance the efficacy of insecticides on porous surfaces merits further investigation.

## Figures and Tables

**Table 1:** MANOVA parameters for main effects and associated interactions for the immediate mortality of *Tenebrio molitor* and *Alphitobius diaperinus* adults between and within exposure intervals (error DF = 160).

Effect	*Tenebrio molitor*			*Alphitobius diaperinus*	
Between exposure intervals					
Source	DF	*F*	*p*	*F*	*p*
Intercept	1	23,297.2	<0.01	18,841.7	<0.01
Dose	1	18.5	<0.01	64.9	<0.01
Food	1	38.6	<0.01	28.4	<0.01
Surface	4	57.0	<0.01	78.2	<0.01
Dose × food	1	0.48	0.49	0.1	0.72
Dose × surface	4	1.0	0.43	3.6	0.01
Food × surface	4	2.7	0.04	1.1	0.37
Dose × food × surface	4	2.1	0.09	1.1	0.37
Within exposure intervals					
Exposure	3	616.5	<0.01	790.7	<0.01
Exposure × dose	3	12.3	<0.01	11.1	<0.01
Exposure × food	3	13.7	<0.01	6.8	<0.01
Exposure × surface	12	25.1	<0.01	26.6	<0.01
Exposure × dose × food	3	0.4	0.74	1.9	0.14
Exposure × dose × surface	12	1.6	0.09	1.8	0.05
Exposure × food × surface	12	2.1	0.02	0.5	0.89
Exposure × dose × food × surface	12	2.4	0.01	2.4	0.01

**Table 2:** Mean immediate mortality (% ± SE) of *Tenebrio molitor* adults exposed to surfaces treated with d-tetramethrin plus acetamiprid for 1 day, 3 days, 5 days, and 7 days. Within each row, means followed by the same uppercase letter are not significantly different (in all cases, DF = 3, 35, Tukey–Kramer HSD test at *p* = 0.05). Within each column, means that are followed by the same lower-case letter are not significantly different (in all cases, DF = 4, 44, Tukey–Kramer HSD test at *p* = 0.05).

Surfaces	1 Day	3 Days	5 Days	7 Days	*F*	*p*
Min/Food						
Plastic	3.3 ± 1.7 Cabc	28.9 ± 3.1 Bab	76.7 ± 4.4 Ab	96.7 ± 1.7 Aa	65.8	<0.01
Glass	8.9 ± 2.0 Ba	54.4 ± 4.4 Aa	95.6 ± 2.4 Aa	100.0 ± 0.0 Aa	43.6	<0.01
Metal	0.0 ± 0.0 Cc	26.7 ± 4.1 Bb	94.4 ± 2.4 Aa	100.0 ± 0.0 Aa	798.0	<0.01
Wood	1.1 ± 1.1 Cbc	14.4 ± 2.4 Bc	64.4 ± 4.1 Ab	87.8 ± 4.0 Ab	80.7	<0.01
Ceramic	6.7 ± 2.4 Bab	47.8 ± 4.0 Aab	94.4 ± 1.8 Aa	100.0 ± 0.0 Aa	43.6	<0.01
*F*	5.6	11.0	17.6	7.1		
*p*	<0.01	<0.01	<0.01	<0.01		
Min/No food						
Plastic	17.8 ± 4.7 Cab	47.8 ± 4.9 Bab	96.7 ± 1.7 Aa	100.0 ± 0.0 Aa	25.0	<0.01
Glass	37.8 ± 5.2 Ca	67.8 ± 4.3 Ba	100.0 ± 0.0 Aa	100.0 ± 0.0 Aa	41.6	<0.01
Metal	1.1 ± 1.1 Cc	41.1 ± 3.9 Bb	95.6 ± 2.4 Aa	100.0 ± 0.0 Aa	213.6	<0.01
Wood	3.3 ± 2.4 Cc	18.9 ± 3.1 Bc	66.7 ± 5.0 Ab	92.2 ± 2.2 Ab	65.0	<0.01
Ceramic	7.8 ± 2.8 Bbc	65.6 ± 5.8 Aa	100.0 ± 0.0 Aa	100.0 ± 0.0 Aa	39.3	<0.01
*F*	15.5	25.5	26.6	11.7		
*p*	<0.01	<0.01	<0.01	<0.01		
Max/Food						
Plastic	13.3 ± 3.3 Ba	64.4 ± 4.1 Aa	96.7 ± 1.7 Aa	97.8 ± 1.5 Aab	27.1	<0.01
Glass	10.0 ± 2.9 Bab	56.7 ± 2.9 Aa	100.0 ± 0.0 Aa	100.0 ± 0.0 Aa	32.7	<0.01
Metal	2.2 ± 1.5 Cb	43.3 ± 3.7 Ba	96.7 ± 1.7 Aa	100.0 ± 0.0 Aa	111.0	<0.01
Wood	2.2 ± 1.5 Cb	18.9 ± 3.9 Bb	73.3 ± 3.3 Ab	94.4 ± 2.4 Ab	52.2	<0.01
Ceramic	7.8 ± 2.2 Bab	56.7 ± 3.7 Aa	100.0 ± 0.0 Aa	100.0 ± 0.0 Aa	43.0	<0.01
*F*	3.6	12.0	34.0	3.6		
*p*	0.01	<0.01	<0.01	0.01		
Max/No food						
Plastic	18.9 ± 4.2 Bab	66.7 ± 3.7 Aa	97.8 ± 1.5 Aa	100.0 ± 0.0 A	17.8	<0.01
Glass	47.8 ± 2.8 Ca	81.1 ± 3.5 Ba	100.0 ± 0.0 Aa	100.0 ± 0.0 A	99.7	<0.01
Metal	2.2 ± 1.5 Cc	45.6 ± 5.0 Bb	98.9 ± 1.1 Aa	100.0 ± 0.0 A	110.7	<0.01
Wood	3.3 ± 1.7 Cc	33.3 ± 3.3 Bb	90.0 ± 3.3 Ab	100.0 ± 0.0 A	73.5	<0.01
Ceramic	13.3 ± 2.4 Bb	71.1 ± 3.1 Aa	100.0 ± 0.0 Aa	100.0 ± 0.0 A	42.3	<0.01
*F*	15.2	24.7	5.8	-		
*p*	<0.01	<0.01	<0.01	-		

**Table 3:** Mean immediate mortality (% ± SE) of *Alphitobius diaperinus* adults exposed to surfaces treated with d-tetramethrin plus acetamiprid for 1 day, 3 days, 5 days, and 7 days. Within each row, means followed by the same uppercase letter are not significantly different (in all cases, DF = 3, 35, Tukey–Kramer HSD test at *p* = 0.05). Within each column, means that are followed by the same lower-case letter are not significantly different (in all cases, DF = 4, 44, Tukey–Kramer HSD test at *p* = 0.05).

Surfaces	1 Day	3 Days	5 Days	7 Days	*F*	*p*
Min/Food						
Plastic	5.6 ± 2.4 Bb	32.2 ± 5.2 Ab	51.1 ± 8.6 Abc	70.0 ± 5.5 Ab	27.8	<0.01
Glass	17.8 ± 2.2 Ca	73.3 ± 2.9 Ba	91.1 ± 2.6 ABa	97.8 ± 1.5 Aa	142.8	<0.01
Metal	0.0 ± 0.0 Dc	32.2 ± 2.2 Cab	58.9 ± 5.6 Bab	77.8 ± 4.7 Aab	798.1	<0.01
Wood	0.0 ± 0.0 Cc	12.2 ± 3.2 Bc	36.7 ± 4.7 Ac	53.3 ± 5.8 Ac	62.3	<0.01
Ceramic	1.1 ± 1.1 Cbc	28.9 ± 4.2 Bb	52.2 ± 4.3 Abc	74.4 ± 4.1 Aab	128.0	<0.01
*F*	25.4	12.7	8.9	10.5		
*p*	<0.01	<0.01	<0.01	<0.01		
Min/No food						
Plastic	8.9 ± 3.5 Bb	58.9 ± 5.6 Aab	77.8 ± 3.2 Aab	93.3 ± 2.4 Aa	31.4	<0.01
Glass	18.9 ± 2.0 Ba	84.4 ± 3.4 Aa	95.6 ± 1.8 Aa	98.9 ± 1.1 Aa	177.4	<0.01
Metal	0.0 ± 0.0 Cc	45.6 ± 5.0 Bab	73.3 ± 4.7 Aab	84.4 ± 4.8 Aa	784.8	<0.01
Wood	2.2 ± 1.5 Cbc	17.8 ± 3.2 Bc	37.8 ± 4.0 ABc	63.3 ± 4.4 Ab	36.1	<0.01
Ceramic	2.2 ± 1.5 Cbc	37.8 ± 4.7 Bb	67.8 ± 3.6 ABb	83.3 ± 4.1 Aa	83.3	<0.01
*F*	13.5	12.7	20.3	13.0		
*p*	<0.01	<0.01	<0.01	<0.01		
Max/Food						
Plastic	14.4 ± 2.9 Ba	63.3 ± 5.3 Aa	85.6 ± 4.4 Aa	97.8 ± 1.5 Aa	31.5	<0.01
Glass	20.0 ± 3.7 Ca	75.6 ± 2.4 Ba	92.2 ± 2.2 Aa	100.0 ± 0.0 Aa	211.1	<0.01
Metal	1.1 ± 1.1 Bb	55.6 ± 5.8 Aa	74.4 ± 4.8 Aa	84.4 ± 4.4 Ab	173.7	<0.01
Wood	1.1 ± 1.1 Db	18.9 ± 2.6 Cb	43.3 ± 3.3 Bb	80.0 ± 2.9 Ab	133.5	<0.01
Ceramic	3.3 ± 1.7 Bb	53.3 ± 3.7 Aa	74.4 ± 2.9 Aa	87.8 ± 3.2 Aab	72.6	<0.01
*F*	17.8	32.9	27.2	8.0		
*p*	<0.01	<0.01	<0.01	<0.01		
Max/No food						
Plastic	15.6 ± 1.8 Cab	77.8 ± 4.3 Ba	96.7 ± 1.7A Bab	100.0 ± 0.0 Aa	195.1	<0.01
Glass	23.3 ± 2.4 Ba	84.4 ± 3.8 Aa	98.9 ± 1.1 Aa	100.0 ± 0.0 Aa	133.4	<0.01
Metal	7.8 ± 2.8 Bbc	77.8 ± 3.6 Aa	92.2 ± 1.5 Aab	97.8 ± 1.5 Aab	40.2	<0.01
Wood	5.6 ± 1.8 Cc	25.6 ± 4.1 Bb	63.3 ± 4.4 Ac	87.8 ± 2.2 Ac	36.3	<0.01
Ceramic	5.6 ± 1.8 Bc	63.3 ± 2.9 Aa	84.4 ± 3.8 Ab	92.2 ± 2.2 Abc	51.5	<0.01
*F*	6.3	31.3	22.1	11.6		
*p*	<0.01	<0.01	<0.01	<0.01		

**Table 4:** Mean delayed mortality (% ± SE) of *Tenebrio molitor* adults exposed for 7 days on untreated dishes, with or without food, after 7 days of exposure on dishes treated with d-tetramethrin plus acetamiprid. Within each row, asterisks indicate significant differences (two-tailed *t*-test at *p* = 0.05). Within each column, means that are followed by the same lower-case letter are not significantly different (two-tailed *t*-test at *p* = 0.05). Where no letters or no asterisks exist, no significant differences were recorded. Where dashes exist, no analysis was performed. Numbers in parentheses denote the number of remaining individuals.

	Min					Max				
Surfaces	Food	No Food	DF	*t*	*p*	Food	No Food	DF	*t*	*p*
Plastic	100.0 ± 0.0 (0)	-	-	-	-	100.0 ± 0.0 (0)	-	-	-	-
Wood	80.6 ± 12.5 (11)	100.0 ± 0.0 (0)	11	1.5	0.16	100.0 ± 0.0 (0)	-	-	-	-
DF	8	-				5	-			
*t*	−1.0	-				-	-			
*p*	0.33	-				-	-			

**Table 5:** Mean delayed mortality (% ± SE) of *Alphitobius diaperinus* adults exposed for 7 days on untreated dishes, with or without food, after 7 days of exposure on dishes treated with d-tetramethrin plus acetamiprid. Within each row, asterisks indicate significant differences (two-tailed *t*-test at *p* = 0.05). Within each column, means that are followed by the same lower-case letter are not significantly different (Tukey–Kramer HSD test at *p* = 0.05). Where no letters or no asterisks exist, no significant differences were recorded. Where dashes exist, no analysis was performed. Numbers in parentheses denote the number of remaining individuals.

	Min					Max				
Surfaces	Food	No Food	DF	*t*	*p*	Food	No Food	DF	*t*	*p*
Plastic	100.0 ± 0.0 (0)	100.0 ± 0.0 (0)	12	-	-	100.0 ± 0.0 (0)	-	-	-	-
Glass	100.0 ± 0.0 (0)	100.0 ± 0.0 (0)	2	-	-	-	-	-	-	-
Metal	91.7 ± 4.2 (3)	81.9 ± 8.7 (3)	14	−1.2	0.24	100.0 ± 0.0 * (0)	50.0 ± 50.0 (1)	7	2.3	0.05
Wood	84.3 ± 6.6 (5)	91.3 ± 4.5 (3)	17	1.0	0.35	92.9 ± 7.1 (3)	100.0 ± 0.0 (0)	14	1.1	0.30
Ceramic	84.4 ± 7.8 (4)	89.3 ± 7.4 (2)	15	0.5	0.65	92.9 ± 7.1 (1)	58.3 ± 20.1 (3)	12	-1.8	0.10
DF	36	27				22	15			
*F*	1.5	0.9				0.4	2.4			
*p*	0.24	0.47				0.75	0.13			

## Data Availability

Data are contained within the article.
